# An alternative method of transperitoneal graft introduction in aortobifemoral bypass surgery

**DOI:** 10.5830/CVJA-2015-011

**Published:** 2015

**Authors:** Yüksel Beşir, Orhan Gokalp, Hasan Iner, Ihsan Peker, Ufuk Yetkin, Koksal Donmez, Levent Yilik, Ali Gurbuz

**Affiliations:** Ataturk Education and Research Hospital, İzmir Katip Celebi University, İzmir, Turkey; Ataturk Education and Research Hospital, İzmir Katip Celebi University, İzmir, Turkey; Ataturk Education and Research Hospital, İzmir Katip Celebi University, İzmir, Turkey; Ataturk Education and Research Hospital, İzmir Katip Celebi University, İzmir, Turkey; Ataturk Education and Research Hospital, İzmir Katip Celebi University, İzmir, Turkey; Ataturk Education and Research Hospital, İzmir Katip Celebi University, İzmir, Turkey; Ataturk Education and Research Hospital, İzmir Katip Celebi University, İzmir, Turkey; Ataturk Education and Research Hospital, İzmir Katip Celebi University, İzmir, Turkey

**Keywords:** aortobifemoral bypass, tunnel, complication

## Abstract

**Introduction:**

Intestinal injury and bleeding, which usually occurs while taking the graft through the transperitoneal tunnel, is one of the most important complications of aortobifemoral bypass surgery. In this study, case reports were examined where, for some reason, the tunneller instrument could not be used to create the transperitoneal tunnel and the tunnelling forceps was used. In some of these cases, the grafts were taken through conventionally and in others an alternative method was used.

**Methods:**

Between 2002 and 2013, the records of 81 patients treated surgically by aortobifemoral bypass for peripheral arterial disease, were investigated retrospectively. In the conventional method, after creating a tunnel with tunnelling forceps, the forceps was re-introduced into the tunnel and the graft was clasped and brought through the tunnel. In the alternative method, a nylon tape was left as a guide in the tunnel while creating the tunnel, and the forceps was not introduced again. The graft was taken through the tunnel with the help of the nylon tape. Patients treated with the conventional method were included in group 1 (*n* = 49) and patients in which the graft was guided with nylon tape were included in group 2 (*n* = 32). The groups were compared peri-operatively.

**Results:**

There were no significant differences between the groups in terms of co-morbidity factors. Extubation time, intensive care length of stay, revision for bleeding, other postoperative complications, and infection and late-term infection rates were similar in the two groups (*p* > 0.05). Hospital length of stay and blood usage were significantly higher in group 1 (*p* < 0.05). Drainage amounts were higher in group 1 but not statistically significant.

**Conclusion:**

Using nylon tape to introduce the graft into the femoral area during aortobifemoral bypass operations was found to be more effective than using the tunnelling forceps.

## Abstract

Aorto-iliac occlusive (AIO) disease is one of the most common forms of arteriosclerosis obliterans (ASO).^1^ The gold-standard treatment of this disease is aortofemoral bypass surgery, according to the Inter-Society Consensus for the Management of Peripheral Arterial Disease (TASC II) study.^1^-^3^

Surgeons have performed this procedure for many years with good long-term results. Vascular damage, bleeding, intestinal damage, ileus, myocardial infarction and renal failure are considered short-term complications. Secondary aorto-enteric fistula, sexual dysfunction, infection, graft thrombosis and anastomotic pseudo-aneurysm may be considered long-term complications.^4^-^6^ Among these complications, vascular damage, intestinal damage and aorto-enteric fistulae usually occur while introducing the graft into the femoral area.

If the tunneller, which was specifically designed for aortofemoral bypass procedures, is not available for some reason, long, blunt-tipped tunnelling forceps are used instead. A nylon tape is taken through the tunnel with the tunnelling forceps after the tunnel is created. Aortic anastomosis is performed after heparinisation.

Connecting the distal ends of the graft to the femoral area is performed in the conventional method by introducing the forceps into the tunnel a second time and pulling the graft through the tunnel. In an alternative method, the nylon tape that is taken through the tunnel with the tunnelling forceps is tied to the graft, which is pulled through into the femoral area. By not introducing the forceps a second time into the tunnel, complications caused by the forceps may be reduced. The results of both methods were analysed for postoperative bleeding, vascular injury and intestinal complications.

## Methods

Between May 2002 and November 2013, 81 patients treated by aortobifemoral bypass (ABFB) via the transperitoneal approach for ASO were examined retrospectively. Parameters such as age, gender, pre-operative co-morbid factors, operative and postoperative data, and postoperative complications and death during follow up of all patients were recorded. Hospital records were used for obtaining the data.

Patients treated with the conventional method were included in group 1 (*n* = 49) and patients in whom the graft was introduced by means of the nylon tape were included in group 2 (*n* = 32). The group results were examined, comparing parameters such as pre-operative data and postoperative complications. Patients who previously had undergone abdominal surgery for any reason and who had had additional non-vascular abdominal surgery were excluded from the study.

The surgical indications were to relieve ischaemic pain, heal ischaemic ulcers, prevent limb loss, improve function and quality of life, and prolong survival, as described in the TASC II consensus. Digital subtraction angiography was performed on all patients to indicate the need for surgery.

Patients who had multiple risk factors and those who had symptoms of coronary artery disease (angina, ischaemic changes on electrocardiography, ischaemia on dipyridamole thallium scintigraphy, or left ventricular wall-motion abnormalities on stress echocardiography) were evaluated by means of pre-operative coronary angiography.

Coronary angiography was performed on three patients in group 1. Two of these patients were treated with angioplasty. In group 2, coronary angiography was performed on four patients and one required angioplasty. None of the patients required surgical intervention for coronary artery disease.

Mean follow-up time was 46.5 ± 27.7 (5–125) months in group 1 and 48.6 ± 29.6 (6–117) months in group 2. All operations were performed under general anaesthesia.

## Surgical procedure in the conventional method

The femoral arteries were explored under the inguinal ligament and appropriate anastomosis sites were examined. The abdomen was explored with upper and lower median incisions. The abdominal aorta was explored and after deciding on the appropriate anastomosis site, the aorta was suspended with nylon tape.

**Fig. 1.**
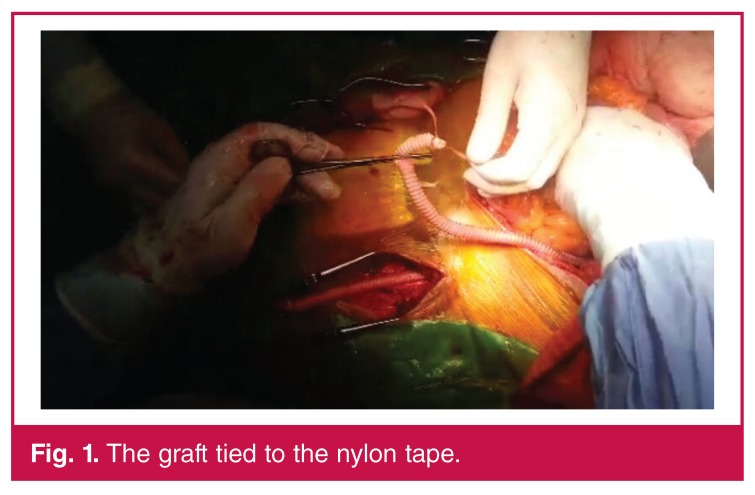
The graft tied to the nylon tape.

Before heparinisation, transperitoneal tunnels were created between the femoral areas and the anastomosis site using a long, blunt-tipped forceps. A long nylon tape was transferred through the tunnel and left inside. After tunnelling, the patient was heparinised and the aortic anastomosis was performed. The nylon tape was then left and the previously created tunnel walls were stretched. Forceps were introduced a second time from the femoral area to the anastomosis site. The distal end of the graft was clasped and pulled through to the femoral area (Fig. 1). The same procedure was applied on the other side. Femoral anastomosis was performed and a drain was left intraperitoneally before closure.

## Surgical procedure with nylon tape

**Fig. 2.**
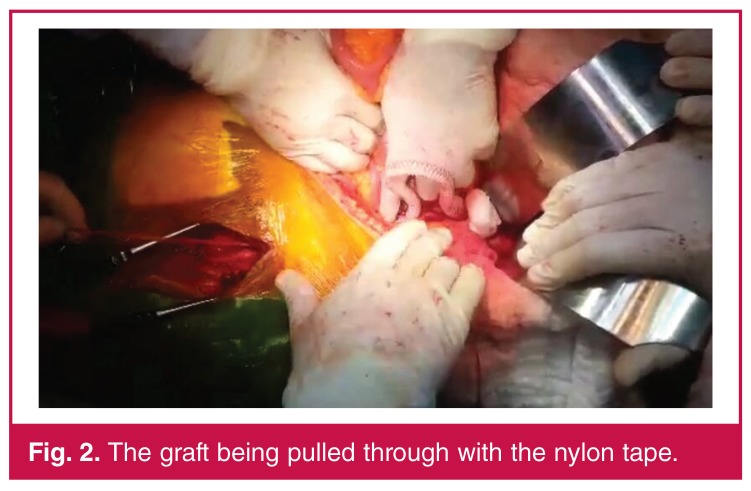
The graft being pulled through with the nylon tape.

**Fig. 3.**
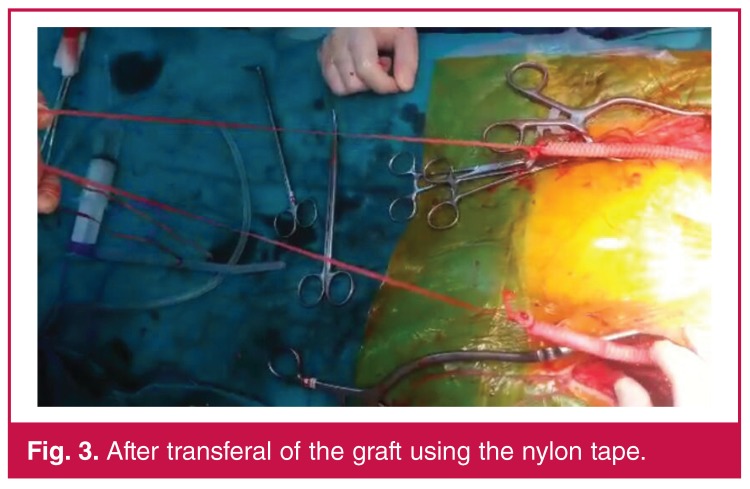
After transferal of the graft using the nylon tape.

The same procedure as in the conventional method was performed up to the aortic anastomosis. The distal ends of the graft were tied to the nylon tape and the aortic clamp was opened. The graft filled with blood. The femoral end of the nylon tape was pulled and the graft was introduced into the femoral area. The same procedure was applied for the other side (Figs 2, 3). Thereafter, the operation was continued as in the conventional method.

## Results

The mean age was 60.98 ± 11.92 (37–92) years in group 1 and 62.88 ± 9.22 (43–81) years in group 2. There was no significant difference between the groups in terms of co-morbidity factors such as diabetes mellitus, coronary artery disease, chronic obstructive pulmonary disease and hyperlipidaemia (*p* > 0.05). Hypertension was significantly higher in group 2 patients (*p* < 0.05). Pre-operative data of both groups are summarised in Table 1.

**Table 1:** Pre-operative data of patients

*Parameters*	*Group 1*	*Group 2*	*p-value*
Age (years)	60.98 ± 11.92	62.88 ± 9.22	0.448
Females	5 (10.2)	2 (6.3)	0.698
Diabetes mellitus, *n* (%)	15 (30.6)	12 (37.5)	0.520
Hypertension, *n* (%)	22 (44.9)	22 (68.8)	0.035
Chronic obstructive pulmonary disease, *n* (%)	7 (14.3)	7 (21.9)	0.377
Hyperlipidaemia, *n* (%)	18 (36.7)	18 (56.3)	0.084

When we compared operative data, we found that operation length was 246 ± 101.62 minutes in group 1 and 231.38 ± 65 minutes in group 2. Despite the operation length being shorter in group 2, it was not statistically significantly different (*p* > 0.05). There was no significant difference between the groups for additional vascular procedures. Operative data of the groups are summarised in Table 2.

**Table 2:** Operative data of patients

*Parameters*	*Group 1*	*Group 2*	*p-value*
Operation length (min)	246.1 ± 101.62	231.38 ± 65	0.490
Additional vascular procedures, *n* (%)	17 (35)	12 (38)	0.789
Embolectomy, *n* (%)	4 (8)	3 (9)	
Endarterectomy, *n* (%)	4 (8)	4 (13)	
Femoropopliteal bypass, *n* (%)	9 (19)	5 (16)	

When we compared postoperative data, there was no significant difference between the groups in terms of extubation time, intensive care length of stay, revision for bleeding, other postoperative complications [such as sexual dysfunction, nerve damage, secondary aorto-enteric fistula (SAEF), ileus, vascular injury or acute renal failure], infection and rehospitalisation for late-term infection (*p* > 0.05). Hospital length of stay and blood usage were significantly higher in group 1 (*p* < 0.05). Postoperative drainage levels were higher in group 1, but not statistically significantly different (*p* > 0.05) (Table 3). Mortality rates were similar in the two groups (*p* > 0.05).

**Table 3:** Postoperative data of patients

*Parameters*	*Group 1 (n = 49)*	*Group 2 (n = 32)*	*p-value*
Extubation time (hours)*	15.07 ± 9.73	16.3 ± 12.61	0.975
Intensive care length of stay (days)*	2.30 ± 1.26	2.25 ± 1.04	0.940
Hospital length of stay (days)*	6.92 ± 1.81	6.09 1.86	0.039
Revision for bleeding, *n* (%)	7 (15)	1 (3)	0.137
Other complications, *n* (%)*			0.731
Ileus	7 (15)	3 (10)	
Inferior vena cava injury	5 (11)	2 (7)	
Acute renal failure	2 (4)	1 (3)	
Postoperative infection, *n* (%)	7 (15.2)	6 (20)	0.588
Postoperative drainage (ml)	490 ± 613	257 ± 318	0.219
Postoperative blood product usage (units)	4.02 ± 2.87	2.04 ± 2.01	0.042
30-day mortality	3 (6.1)	2 (6.3)	0.981

In group 1, three patients died, two because of multiple organ failure and one because of myocardial infarction at late term. In group 2, two patients died, both because of multiple organ failure.

## Discussion

The gold-standard treatment for aorto-iliac occlusive disease is ABFB. This procedure has been performed for many years with good long-term results. Despite many modifications for reducing complications (retroperitoneal approach and minimally invasive approach), the transperitoneal approach is still the most widely used technique.^1^,^7^

Many studies have proved that the minimally invasive approach has advantages for cardiac risk, postoperative complications and postoperative ileus, but a randomised, prospective study did not prove any significant advantage over the conventional technique.^1^ The minimally invasive approach is advised for patients with previous abdominal surgery or co-morbidities, and the elderly.

In this study, we preferred the conventional approach. There were some complications of ABFB with the conventional approach, which may have been specific to the surgery, such as SAEF, vascular injury, bleeding, intestinal injury, ileus, myocardial infarction, renal failure, sexual dysfunction, infection, graft thrombosis, anastomotic pseudo-aneurysm (which may differ in different abdominal approaches), or non-specific complications such as myocardial infarction, pulmonary complications and renal dysfunction.^1^,^4^-^7^

Chiu *et al.* revealed that, although there were different rates of complications in different series, rates were approximately 16% in their review.^8^ The rates ranged between 0 and 11% in other reviews.^8^-^12^

Postoperative bleeding is a common early complication and causes re-operation in 1–2% of patients.^13^ Inadequate control of bleeding, anastomotic technique, intra-operative use of heparin, and dilutional coagulopathy occurring after blood loss have been shown to be the most common causes of this complication.^13^

Another complication in the postoperative period is acute renal failure. Declamping and lack of fluid balance are thought to be the cause of this complication.^13^ Mortality rates in our study were 6.15% in group 1 and 6.3% in group 2, which was similar to that in the literature.

Complication rates (excluding death) were 15% in group 1 and 10% in group 2. Acute renal failure was found in only one patient in group 2. Bleeding requiring re-operation was found in seven patients in group 1 and one in group 2. SAEF, rarely seen in our series but commonly encountered in the literature, was not observed in any of our patients. Inferior vena cava injury, termed vascular injury, was seen in two patients in group 1 but none in group 2.

We believe some of the complications seen in other cases may have been associated with manipulation by the tunneller during surgery. A study by Luo and colleagues, comprising a case report accompanied by a literature review, is one of the studies supporting our theory.^14^

In our study, the tunneller was not used and forceps were introduced into the tunnel a second time in the conventional method. Postoperative bleeding amounts were higher but not statistically significant in the conventional method. Peri-operative blood usage was significantly higher in the conventional method. Although it was not statistically significant, ileus rates were higher in the conventional method. This situation may have been related to longer hospital stay due to bleeding.

Our study has some limitations. Group sizes were particularly small and graft patency data were not obtained for all patients.

## Conclusion

Some complications of ABFB, which are directly related to the surgery, may be avoided, especially in cases where the tunneller is not used. Nylon tapes that are left in the tunnel while creating it may be used to introduce the distal end of the graft into the femoral area. This alternative method must be kept in mind as it has lower complication rates than the conventional method.
